# Are You on My Wavelength? Interpersonal Coordination in Dyadic Conversations

**DOI:** 10.1007/s10919-019-00320-3

**Published:** 2019-10-15

**Authors:** Joanna Hale, Jamie A. Ward, Francesco Buccheri, Dominic Oliver, Antonia F. de C. Hamilton

**Affiliations:** 1 Institute of Cognitive Neuroscience, UCL, Alexandra House, 17 Queen Square, London, WC1N 3AZ UK; 2 Computing Department, Goldsmiths University of London, London, UK

**Keywords:** Conversation, Social coordination, Motion capture, Mimicry, Synchronization, Nonverbal

## Abstract

Conversation between two people involves subtle nonverbal coordination in addition to speech. However, the precise parameters and timing of this coordination remain unclear, which limits our ability to theorize about the neural and cognitive mechanisms of social coordination. In particular, it is unclear if conversation is dominated by synchronization (with no time lag), rapid and reactive mimicry (with lags under 1 s) or traditionally observed mimicry (with several seconds lag), each of which demands a different neural mechanism. Here we describe data from high-resolution motion capture of the head movements of pairs of participants (*n *= 31 dyads) engaged in structured conversations. In a pre-registered analysis pathway, we calculated the wavelet coherence of head motion within dyads as a measure of their nonverbal coordination and report two novel results. First, low-frequency coherence (0.2–1.1 Hz) is consistent with traditional observations of mimicry, and modeling shows this behavior is generated by a mechanism with a constant 600 ms lag between leader and follower. This is in line with rapid reactive (rather than predictive or memory-driven) models of mimicry behavior, and could be implemented in mirror neuron systems. Second, we find an unexpected pattern of lower-than-chance coherence between participants, or hypo-coherence, at high frequencies (2.6–6.5 Hz). Exploratory analyses show that this systematic decoupling is driven by fast nodding from the listening member of the dyad, and may be a newly identified social signal. These results provide a step towards the quantification of real-world human behavior in high resolution and provide new insights into the mechanisms of social coordination.

**Electronic supplementary material:**

The online version of this article (10.1007/s10919-019-00320-3) contains supplementary material, which is available to authorized users.

## Introduction

Face-to-face conversation between two people is the fundamental basis of our social interaction (Clark 1996) and is of increasing interest in both clinical (Ramseyer and Tschacher 2011) and neuroscientific (Schilbach et al. 2013) research. Despite this, we have only limited data on the precise timing and patterns of nonverbal coordination which are found in real-world conversation behaviors. Early work coding actions from videos shows that people tend to move coherently with one another, which has been described as mimicry or synchrony (Bernieri and Rosenthal 1991). Behavioral studies suggest that this coordination acts as a ‘social glue’ (Lakin et al. 2003) that predicts the success of a negotiation or meeting (Pentland 2008).

The initial aim of the present study was to use high precision motion capture to record the head motion of dyads in conversation, and then perform an advanced analysis to reveal the time course of mimicry. In particular, we aimed to resolve a conflict between behavioral reports of mimicry with time lags (e.g., 2–10 s) (Leander et al. 2012; Stel et al. 2009) and studies of the neurophysiological mechanisms of mimicry in terms of mirror neurons which imply a much shorter time lag (e.g., 350 ms) (Heyes 2011; Iacoboni et al. 1999). Our data address this issue but also reveal novel patterns of lower-than-chance coherence, or ‘decoupled’ behavior going beyond simple mimicry or synchrony. We conducted a pre-registered study to confirm our results, and report here a detailed analysis of head motion coherence in dyads, as found in a structured conversation task.

### Background

It is increasingly recognized that neuroscience needs to examine real-world behavior in detail (Krakauer et al. 2017), and that social neuroscience needs to understand the interactions of two real people rather than just studying solo participants watching videos of people (Schilbach et al. 2013). A long tradition of research into human interpersonal coordination and conversation describes patterns of nonverbal synchrony and mimicry as key behaviors in dyadic interactions. From early work (Condon and Ogston 1966; Kendon 1970) to the present (Chartrand and Bargh 1999; Ramseyer and Tschacher 2011), it is clear that people tend to move their heads and bodies in coordination with each other. Untrained observers can perceive a gestalt of coordination in videos of conversations, and rate higher coordination in genuine interactions compared to ‘pseudo interactions’ where interaction partners from different videos were randomly paired together to make it look as though they were having a real interaction (Bernieri 1988). Global ratings of conversational coordination also predict interpersonal liking (Salazar Kämpf et al. 2018) but curiously, specific mimicry behaviors could not easily be identified in these participants. Such studies clearly demonstrate that *something* important and interesting is happening in live dyadic interactions, but it is less clear exactly what this *something* is, and what mechanisms might support it.

Interpersonal coordination arises across a range of different body parts and modalities, including coordination of head movement, posture, gesture and vocal features (Chartrand and Lakin 2012). The present paper focuses only on head movements, because mimicry has been reported here both in behavior and virtual reality (Bailenson and Yee 2005). Two major types of coordination can also be distinguished, which we refer to as ‘mimicry’ and complementary behavior. Here, mimicry refers to events where two people perform the same action (regardless of timing), while complementary behavior refers to events where two people perform different actions. Within the domain of head movements, mimicry might occur when one person looks downwards and the other looks down shortly after, while complementary behavior might occur if one person remains still while the other nods in agreement. In this latter case, the head movement (nodding) is being used as a conversational back-channel (Kendon 1970). While both these types of movements can occur, the ‘social glue theory’ claims that mimicry movements have a somewhat special status and can lead to liking and affiliation. Thus, the primary goal when we set up this study was to identify the features of naturalistic mimicry movements and understand more about the cognitive processes that can produce this behavior.

**Fig. 1:**
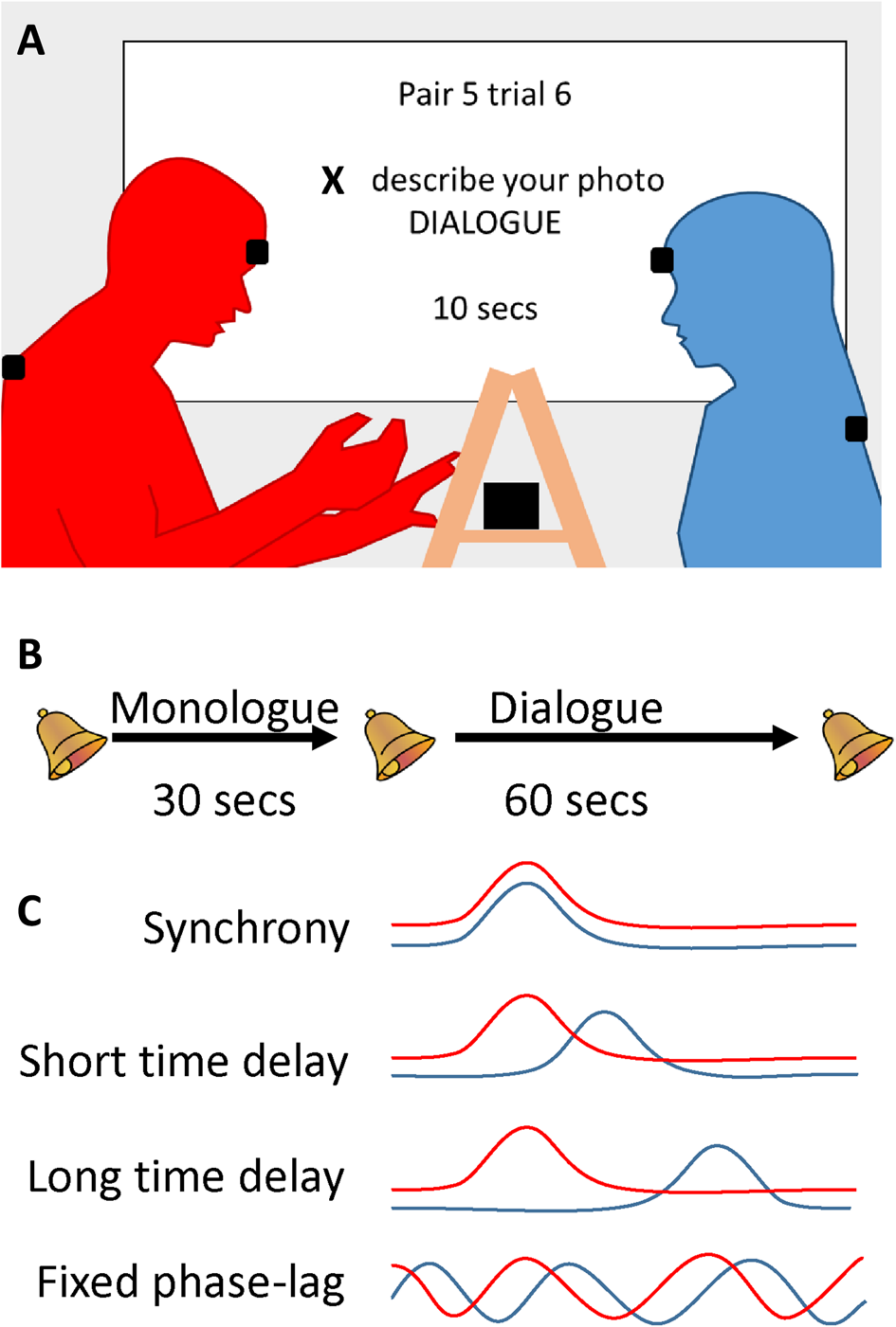
**a** Data collection. Two participants sat approximately 1.5 m apart on small stools. A wooden frame between the participants held the picture (for description) and the Polhemus transmitter box. Polhemus motion sensors were placed on the upper back and forehead of each participant. A projector screen beside the participants provided instructions, timing cues, and video synchronization. **b** Trial structure. Each trial had 30 s of monologue by one participant, designated Leader, followed by 60 s of dialogue with the Follower, marked by beep sounds. Participants completed 16 trials, alternating turns as Leader or Follower. **c** Possible patterns of behavior. Four possible patterns of interpersonal coordination are illustrated schematically

A detailed understanding of mimicry in dyadic coordination is important because it can constrain our theories of the mechanisms underlying this coordination. For a real-time coordination between two people, there are at least four different possible mechanisms which have been proposed with different timing properties (Fig. 1c). First, a predictive mechanism would allow perfect synchrony between two people (0 ms lag) with both people making the same movement at the same time. This is seen in musical coordination (Konvalinka et al. 2010), and performing in synchrony with other people can lead to improvements in memory (Macrae et al. 2008) and affiliation (Hove and Risen 2009). Second, mimicry could arise via a simple visuomotor pathway (Heyes 2011), likely implemented in the human mirror neuron system (Iacoboni et al. 1999), whereby perceived actions automatically activate a corresponding motor response. Lab studies show that such responses can occur as fast as 350 ms after a stimulus (Brass et al. 2001) so we suggest that 400–1000 ms is a plausible time range for reactive mimicry in natural conversation. Third, mimicry might happen on much longer timescales of 2–10 s, as reported in studies observing real-world behavior (Leander et al. 2012; Stel et al. 2009). These longer time delays imply that a short-term memory mechanism must be involved, in addition to the mirror neuron system, in order to maintain a representation of the observed action until it is performed. Finally, behavioral coordination might be governed by a particular pattern of phase relationships, such as in-phase movements (oscillating together) being preferred to anti-phase movements (oscillating apart) (Oullier et al. 2008). If the same phase-relationship between the two people is found across a range of motion frequencies, this implies a cognitive mechanism tuned to maintaining a preferred phase-relationship (e.g., fast mimicry of fast actions and slow mimicry of slow actions). Though the neural mechanism of a fixed phase pattern is unclear, some previous papers focus on understanding phase relationships in conversation behavior which implies that phase is a key parameter (e.g., Fujiwara and Daibo 2016; Issartel et al. 2014). Our study tests if this is true. Overall, understanding the timing of social coordination and the frequency at which it occurs will help us understand the computational mechanisms which implement this coordination.

To record nonverbal behavior in conversations, studies have traditionally used video, which can be coded by trained observers to detect specific behaviors. However, hand-coding of videos in these studies may introduce biases (Cappella 1981) and limits the amount of data which can be processed as well as its resolution (Grammer et al. 1998). More recently, researchers have begun to use automated methods to calculate motion energy or other parameters of behavior from video (Fujiwara and Daibo 2016; Paxton and Dale 2013; Ramseyer and Tschacher 2010; Schmidt et al. 2012), but resolution is still limited to the pixels which change in a flat image. Motion capture provides much higher resolution (Feese et al. 2011; Poppe et al. 2013), so we use this method here to record the head movements of participant dyads engaged in structured conversations. The motion capture system we use provides a rich dataset of each participant’s head angle at 60 time points per second (60 Hz). Head yaw (left–right turning angle), pitch (up-down nodding angle), and roll (shoulder-to-shoulder tilting angle) are recorded as separate signals of head motion at each timepoint (see Fig. 3 for diagrams).

To extract meaning from the motion capture dataset, each head motion signal (yaw, pitch, or roll) is converted to a time-frequency spectrum using wavelet analysis. This wavelet representation captures information on a range of constituent frequencies of the signals across the time points recorded during the interaction. From this, the cross-wavelet coherence between the wavelets of two different people is calculated to give a measure of the coordination between their movements for a given motion frequency and a given timepoint (Fujiwara and Daibo 2016; Issartel et al. 2014). Proof of concept studies using wavelet methods have found that coordination exists during musical improvisation (Walton et al. 2015) and telling knock–knock jokes (Schmidt et al. 2014). These studies suggest that coordination occurs at multiple timescales (Schmidt et al. 2014) and frequencies (Fujiwara and Daibo 2016) with less coherence at high motion frequencies close to 4 Hz. However, this previous work did not isolate the axes of movement (pitch, yaw, roll) from overall motion energy, nor did it perform a robust comparison between real dyads and pseudo dyads.

#### The Present Study

In the present study, we recorded movements from pairs of participants (dyads) engaged in a picture description task (Fig. 1). This task has been used previously in behavioral (Chartrand and Bargh 1999) and motion capture studies (Shockley et al. 2003). In each trial of our task, one participant had the role of Leader, holding a picture of a complex scene while the other had the role of Follower (Fig. 1). Each trial comprised 30 s of monologue where the Leader describes the picture and the Follower remains silent, followed by 60 s of dialogue where both participants can speak. We recorded the position and rotation of motion sensors on each participant’s head and upper back at 60 Hz. As wavelet analysis is a relatively new technique in social cognitive research, the present study consisted of a pilot phase and a final phase. The pilot phase (*n* = 20 dyads) was highly exploratory and the data were used to develop and test our analysis algorithms. We then pre-registered our methods (Hale and Hamilton 2016) and collected a second independent dataset (*n* = 31 dyads), which we report here. Our analysis uses wavelet coherence measures to quantify the interpersonal coordination at different frequency bands from 0.2 to 8 Hz for the head pitch (nodding) signal. We chose to focus only on the head-nodding data based on our pilot study which showed similar results across the pitch, roll, and yaw signals but that the pitch (nodding) results were slightly stronger. By specifying a priori that we will focus only on nodding (in our pre-registration document) and maintaining this focus, we reduce the problem of multiple comparisons and are able to obtain robust results. Our aim was to precisely characterize the frequency, phases and patterns of head motion coordination in a conversation, and to test cognitive models of this coordination.

## Method

### Participants

We recruited 31 dyads from a local mailing list. Participants were paired up as dyads on the basis of their availability and preference for the same time slot. In line with our pre-registered plan, we excluded data from dyads who met any of the following criteria:

1. Participants knew each other before the study
2. Motion data were not recorded due to technical failure of the equipment or task software
3. Motion sensor(s) moved or fell off during the study
4. More than 50% of their data are missing or not suitable for wavelet analysis

Data were excluded from one dyad based on criteria 1 (familiarity), two dyads on criteria 3 (sensors fell off) and two dyads on criteria 4 (missing data). After these data exclusions, we report results from 26 dyads (*M*_age_ = 22.3 years, *SD*_age_ = 2.9 years). There were 16 same-sex dyads and 10 mixed-sex dyads (34 female and 18 male participants). All participants gave written consent and were paid £7.50 for 1 h. The study received ethical approval from the UCL Research Ethics Committee and was performed in accordance with the 1964 Declaration of Helsinki.

### Lab Setup

The room was set up with two wooden stools for the participants, facing each other at a distance of approximately 1.5 m. Between the stools, a wooden frame held a Polhemus transmitter device (Polhemus Inc., Vermont), which generated an electromagnetic field of approximately 2 m diameter around the participants. A projector screen to the side of the participants showed instructions throughout the session, and audio speakers behind the projector provided audio cues. A curtain separated participants from the experimenter, who remained in the room but did not interact with participants during the experiment and could not be seen.

A Polhemus magnetic motion tracking device recorded the head and torso movements of each participant at 60 Hz. One sensor was fixed on the participant’s forehead using a Velcro cloth band. Another sensor was attached to the participant’s upper back using microporous tape. Both participants wore a lapel microphone and their voices were recorded on the left and right channels of a single audio file. A video camera mounted on a tripod recorded the session, offering a clear view of the participants’ seated bodies. We used Vizard software (WorldViz, California) to display instructions on the projector screen, trigger audio cues, and record data.

### Procedure

Pairs of participants arrived and gave informed consent to take part. They were fitted with motion sensors and microphones, and randomly assigned to be the ‘X’ participant or ‘Y’ participant. These labels were used to distinguish dyad members during the experiment and in the recorded data. Participants completed one practice block followed by four experimental blocks. Each block was made up of a short language task, followed by four trials of the picture description task. The language task was a social priming task as used in Wang and Hamilton (2013), which was designed to activate prosocial or antisocial concepts in the participants. Both participants completed the same type of priming (prosocial or antisocial) with different exemplars. As the priming had no discernible impact on behavior in the task, we do not discuss it further.

The picture description task was based on Chartrand and Bargh (1999) with a more controlled time-course to allow averaging between trials. On each trial, one participant held a picture of a complex social scene, taken from the National Geographic website and printed on heavy card. The participant with the picture was the Leader for that trial, and had 30 s to describe the picture to the Follower, while the follower could not speak (monologue period). When a beep signaled the start of the 60 s dialogue phase, the Follower was allowed to ask questions and the dyad could converse freely about the picture. Audible beeps indicated the start of the trial, the start of the monologue section, and the end of the trial. A timer on the projector screen also counted down the time left in the monologue and dialogue sections. Thus, each picture description trial lasted 90 s with a fixed time structure in all trials. Each dyad completed 16 trials, alternating between Leader and Follower roles (Fig. 1).

At the end of the study, participants individually completed six Likert ratings about the quality of their interaction, e.g., smoothness and rapport (see Hale and Hamilton 2016 for items). Questionnaire data were checked to ensure there were no outliers (e.g., dyads who report hating each other), but were not analyzed further. Finally, participants were asked to write down what they thought was the purpose of the study and were debriefed and paid by the experimenter.

### Analyses: Pre-processing

Raw data were recorded as x-y-z position coordinates and yaw-pitch-roll angle signals from 4 motion sensors—a head sensor and torso sensor of each participant, giving 24 data signals at 60 Hz for each 90 s trial. Pre-processing of the data was performed in Matlab. We trimmed data by discarding the first 100 time points (1.7 s of the trial), all time points after the 5250th point (87.5 s into the trial; note that we originally specified 5300 in our pre-registration, but some trials were shorter than 5300 data points). This removes irregularities at the start or end of trials and ensures that all trials are the same length. We corrected for circularity in the rotation data, to deal with cases where a change in orientation from − 355° to 5° appears to Matlab like a large change rather than only a 10° movement past zero. To correct for small inaccuracies in timing, we resampled the data using a polyphase anti-aliasing filter (using the Matlab resample function). This was to ensure that we had a uniform fixed sample rate that exactly matched the number of expected data points. Each data signal was then de-trended by subtracting the mean value. Finally, we applied a 7th order Butterworth low pass filter with cut-off frequency of 30 Hz to reduce noise.

**Fig. 2:**
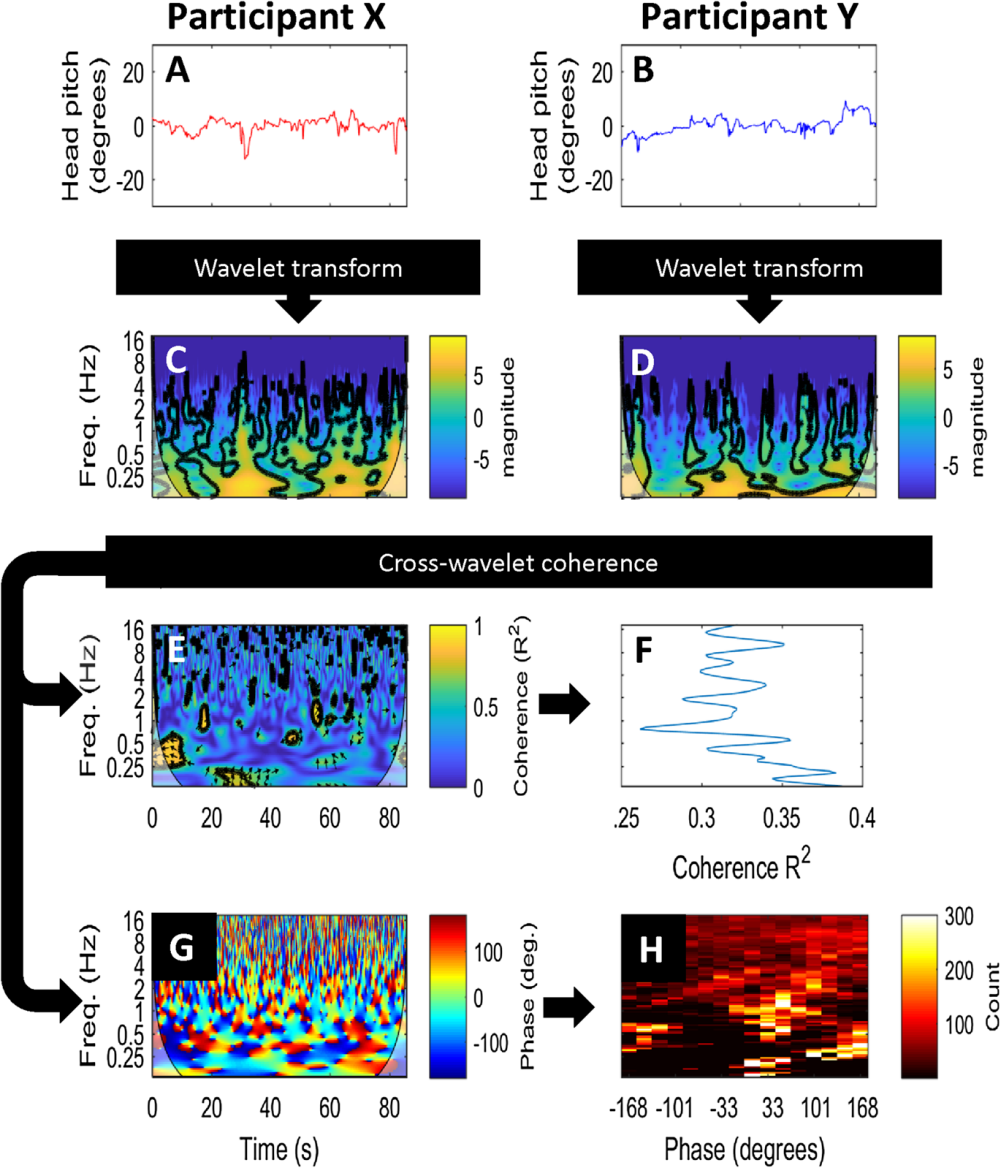
Data analysis methods. For each trial of true interaction, the head motion traces for the two participants (**a**, **b**) were subject to a wavelet transformation giving **c** and **d**. The cross-wavelet coherence was calculated to give coherence (**e**) and phase angle (**g**) across the frequency spectrum for all time points during the trial. The coherence (**e**) was averaged over time (**f**) and then over all dyads to obtain the average coherence across the frequency spectrum in real interactions. The phase angles (**g**) were binned by angle and frequency (collapsing over time) (**h**) and then averaged over all dyads. To calculate coherence and phase angle in pseudo-interactions, the same procedure was used with mismatched data replacing the inputs **a** and **b**

Of the 24 data signals collected, the final analysis focused primarily on head pitch because this was the most informative signal in our pilot analysis. Other head signals are presented in supplementary information and Hale’s thesis (Hale 2017). We conducted several different analyses, which we describe in relation to the pipeline shown in Fig. 2.

### Wavelet Coherence Analysis

The raw data signals for the X and Y participants (Fig. 2a, b) were subject to a wavelet transform and then the cross-wavelet coherence was calculated, using the Matlab toolbox from Grinsted et al. (2004) with default parameters (Morlet wavelet, *w* = 6), according to our pre-registration. Note that other parameters (Morlet wavelet, *w* = 8) are suggested by (Issartel et al. 2007), but when applied to our dataset did not change the results. To prevent edge effects from the start and stop times of the interaction (as well as the times surrounding the prompted switch from monologue to dialogue), we discounted coherence results from the so-called ‘cone of influence’ (COI) (i.e., the pale areas in the corners of Fig. 2c, d, e). In a very small minority of trials (mainly those with a single very jerky movement), the wavelet toolbox is unable to calculate the wavelet transform. Such trials were excluded from all analyses and reported as missing data.

Next, we averaged the cross-wavelet coherence over the time course of each trial to obtain a simple measure of the frequency of coherence without regard to timing within a trial (Fig. 2f). We truncated the wavelet output to frequencies from 0.2 to 8 Hz, resulting in a 1 × 89 vector of coherence values for 89 frequency bins. This is a smaller frequency range than specified in our pre-registration, because we found that data above 16 Hz could have been contaminated by dropped frames (affecting 2.7% of data points). Also, there were not enough data below 0.2 Hz (5 s period) to give a meaningful result, because with the COI removed, less than 10 s of useable monologue data remains below 0.2 Hz. Thus, our final output from the wavelet analysis is a coherence vector for each trial of each dyad, giving values of interpersonal coherence across frequencies from 0.2 to 8 Hz.

### Wavelet Coherence in True Trials Compared to Pseudo-trials

To fully quantify the levels of interpersonal coherence we observe in our data, we need to compare this to a null dataset where coherences is not present. We can do this by calculating the coherence present in pseudo-trials, where data from two different interactions are entered into the algorithm as if they were the X and Y participants (Bernieri and Rosenthal 1991; Fujiwara and Daibo 2016). Previous studies using this approach created pseudo trials by mixing datasets from different participants. We adopted a stricter approach where we create pseudo-trials using data from trials of the same dyad with the same leader-follower assignment. For example, if trials 1, 3, 7, and 9 have X as leader and Y as follower, the true trials are 1–1; 3–3; etc., while the pseudo trials are 1–3; 7–3; etc. Thus, our pseudo trials had the same general movement characteristics (e.g., overall signal power) as our real trials, and differed only in that the real trials represent a genuine live social interaction. This strict definition of pseudo trials within-pair and within-role gives the strongest possible test that any differences between the coherence levels in the real and pseudo pairs must be due to the live interaction and not to differences in participants or roles. We carried out wavelet analysis on the pseudo dataset using the same pipeline as above, and thus calculated the overall coherence values for all possible pseudo-trials of each dyad.

**Fig. 3:**
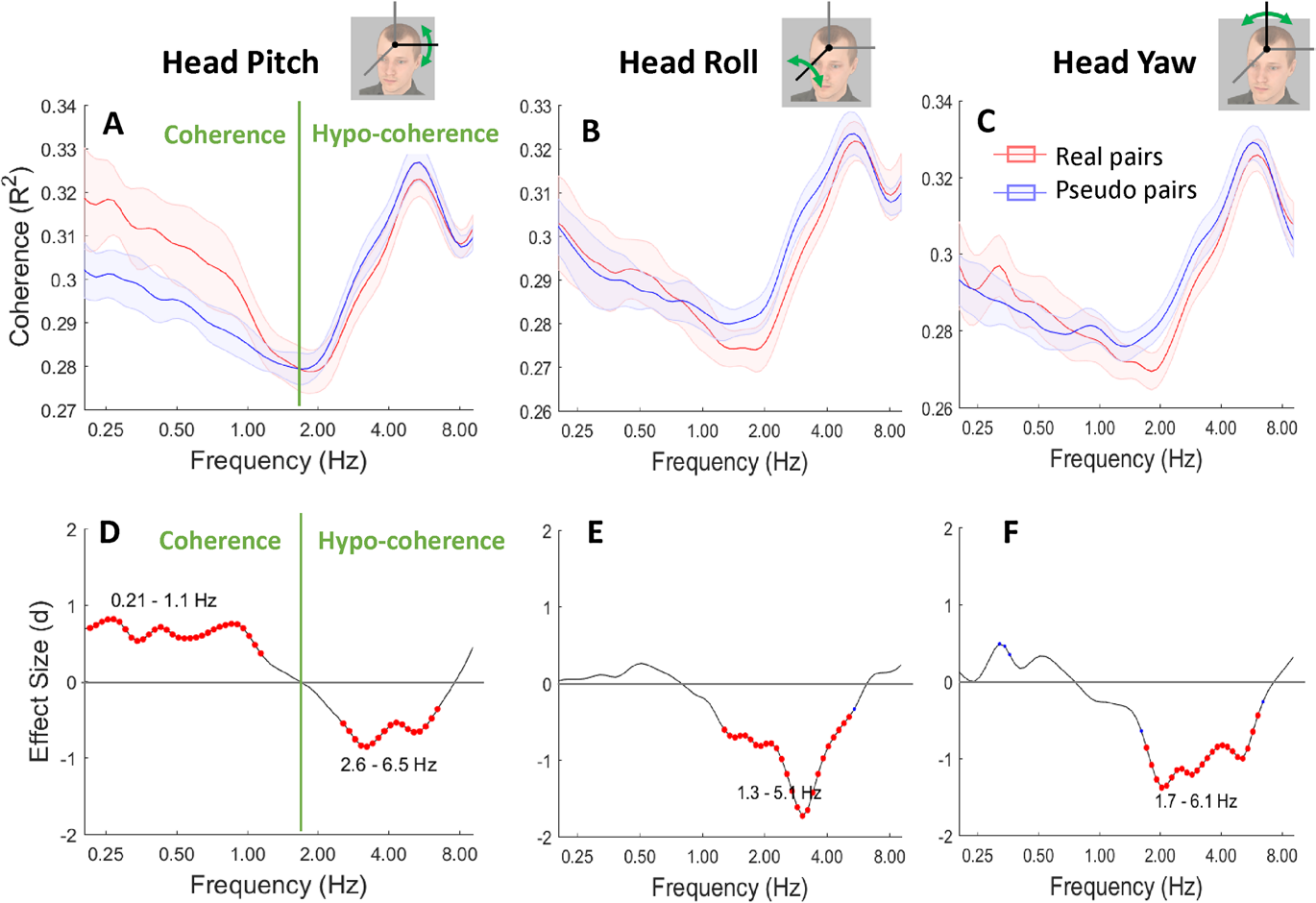
Wavelet coherence results. **a** The mean and standard error of coherence for real trials (red, upper line on the left) and pseudo trials (blue, lower line on the left) at all motion frequencies. Across the same frequencies, **d** the effect size for a t-test on the difference between real and pseudo pairs, with red dots indicating results that pass a *p* < .05 FDR significance threshold. The green vertical line on each panel indicates where we interpret the results as coherence at low frequencies (left of plot; coherence was greater in real than pseudo trials) and hypo-coherence at high frequencies (right of plot; coherence was greater in pseudo than real trials). **a** and **d** show the pre-registered analysis of head pitch. Equivalent exploratory plots for roll and yaw are shown in **b**, **c**, **e**, and **f** (Color figure online)

To implement group-level comparisons, we compare each dyad’s real trial coherence to that dyad’s pseudo trial coherence, using paired t-tests for each of the 89 frequency bins between 0.2 and 8 Hz. We correct for multiple comparisons using FDR (Benjamini and Hochberg 1995) and plot the results both in terms of mean coherence levels and effect sizes for head pitch, yaw, and roll (see Fig. 3).

### Additional Analysis

In addition to the preregistered analyses described above, we conducted several exploratory analyses. These allowed us to test if the effects are similar across monologue and dialogue, and to test if the effects we see are driven by global differences in signal power. The results we find revealed different patterns of behavior at high frequencies and low frequencies. To further explore these, we built a ‘fast nod detector’ which can encode the presence of high-frequency nodding, and tested how this relates to who is speaking or not speaking. We also conducted a detailed analysis of the phase relationships between leader head movement and follower head movement for low-frequency movements. Detailed methods for all these analyses are presented in supplementary information (see Supplementary Information), and the key results are available below.

## Results

### Novel Patterns of Cross-wavelet Coherence of Head Movements in Real versus Pseudo Interactions (Pre-registered Analysis)

Each dyad completed 16 trials of the conversation task, 8 with person “X” as the leader, and 8 with “Y” as the leader. For each trial and each head motion signal (yaw, pitch, roll), we calculated the wavelet transform and then the wavelet coherence (Fig. 2). Levels of coherence in head pitch (nodding) at each motion frequency are shown in Fig. 3a, with the mean coherence in real trials (red) and pseudo trials (blue). To assess the difference in cross-wavelet coherence between real and pseudo interactions, we performed t-tests at each frequency (89 tests). Figure 3d shows the effect size (Cohen’s *d*) for the difference between head pitch coherence in real versus pseudo interactions. Red dots indicate frequencies where there was a significant difference between real data and pseudo data, with FDR correction for multiple comparisons (Benjamini and Hochberg 1995).

Two patterns are noticeable in these data. First, there is greater coherence in real pairs than pseudo pairs at low frequencies (0.21–1.1 Hz). We examine the phase relationships and dominant time lags in this frequency range below. Second, there is a marked dip in coherence in the real pairs at higher frequencies (2.6–6.5 Hz), compared to pseudo pairs. This suggests that coherence in this frequency range is lower-than-chance, a phenomenon we refer to as hypo-coherence. The hypo-coherence we found at high frequencies was unexpected, given the strong focus on moving together in previous literature. When we first saw this pattern in our pilot data (Hale 2017), we worried about its robustness and so conducted a replication study with a larger sample size and a pre-registered data analysis pathway (Hale and Hamilton 2016). It is the pre-registered results we report here (Fig. 3a, d). These data show that both the coherence at low frequencies and the hypo-coherence at high frequencies are reliable and replicable. An exploratory analysis of head-roll and head-yaw shows similar hypo-coherence at high frequency in these data too (Fig. 3b, c). Below we describe further exploratory analyses to understand both the coherence of head pitch at low frequency and the hypo-coherence in pitch at higher frequencies.

### Exploring Coherence in Head Pitch at Low Motion Frequencies

Our data revealed a positive coherence between dyads at 0.2–1.1 Hz in head pitch (nodding). These frequencies are commonly linked to mimicry behavior, and therefore positive coherence in this range is a plausible signature of the mimicry of one participant’s head movement by the other. Note that positive coherence does not mean that both participants mimic all the time, but rather that one performs a head movement action and then the other moves in the same direction within the time window of the wavelet analysis. We conducted several analyses to check the generality of this low-frequency coherence. While the effect is robust in the pitch data (Fig. 3a), we did not see the same pattern in the roll or yaw data (Fig. 3b, c). We can also split our data up to examine the monologue part of the trial separately from the dialogue part (see Supplementary Information Figure S1). We find that motion coherence at low frequencies is present in both the monologue and the dialogue sections of the conversation, indicating that this effect is not altered by this manipulation of context, but seems to be a general phenomenon.

**Fig. 4:**
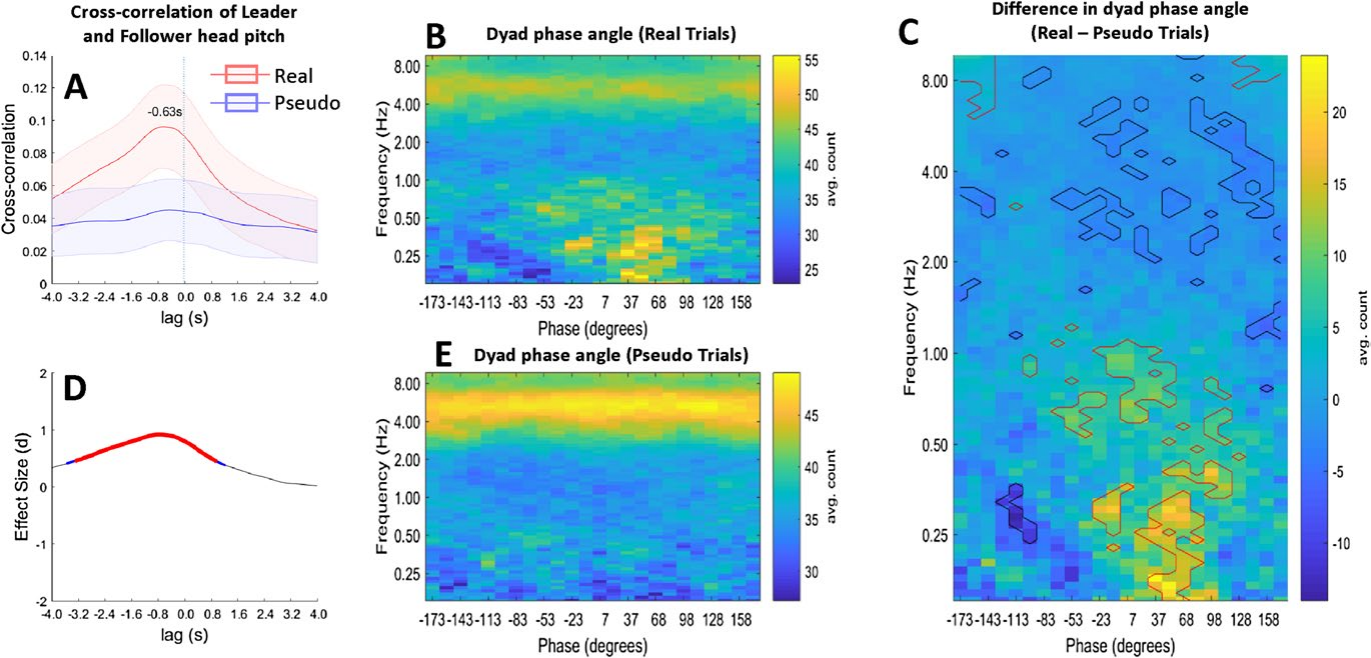
Phase angle analysis. **a** Average cross-correlation of head pitch between Leader and Follower participants across a whole trial, for time delays of − 4–4 s. There is a peak at − 0.63 s, indicating that Followers match Leaders with around a 600 ms time delay. **d** The Cohen’s-d effect size for the difference in cross-correlation between real and pseudo trials, for time delays of − 4–4 s. Red dots indicate significant t-tests that pass a *p* < .05 FDR significance threshold. **b** (real trials) and **e** (pseudo trials) show the average phase angle within a dyad for all time points across the trial (y-axis) and all motion frequencies (x-axis). Panel C shows the difference between phase angles in real trials (**b**) and pseudo trials (**e**). Regions outlined in red show the phase angles and frequencies where there was significantly greater coherence in real than pseudo trials (a positive effect), indicating that the Follower lags the Leader by around 30–90° of phase. Regions outlined in blue show where there was a significant negative effect (Color figure online)

To test our cognitive models, a key question concerns the time lags or phase relationships between participants—do they move synchronously, with a fixed time lag or with a fixed phase relationship? Our first analysis of this question examined the cross-correlation between the Leader’s head pitch and the Follower’s head pitch over each whole trial. Leader to Follower cross-correlation was calculated across a range of different time delays (− 4–4 s) for real and pseudo trials. This range was chosen to cover the likely time delays. These results are averaged and shown with standard error (Fig. 4a), and with Cohen’s-d effect size (Fig. 4d) for the comparison between the real and pseudo conditions. We find that real trials have greater cross-correlation than pseudo trials across a range of time delays from − 3 to 0.8 s, with a peak at − 0.6 s. This implies that the Follower tends to match the head movements of the Leader with around a 600 ms delay.

We also examined the phase relationship between the two participants for the regions of the frequency spectrum with positive coherence. That is, for each wavelet coherence plot of each trial (Fig. 2e), we calculated the phase relationship between leader and follower at every time-frequency point (see supplementary information). Figure 2 gives an example of this analysis; we calculated relative phase from Leader to Follower for each point in the wavelet coherence plot (Fig. 2g) and then collapsed over time to obtain a phase-frequency histogram in which phase-angle counts are represented in 24 phase bins (Fig. 2h). We averaged the phase-frequency histograms over trials and participants for both real trials and pseudo trials. Figure 4 shows the average phase plot over all trials for real (Fig. 4b) and pseudo pairs (Fig. 4e) with the difference between these in Fig. 4c. The red circled areas of positive significance (*p* < .05 in a paired *t* test, *df* = 25) show that coherence occurs in the 0.2–0.5 Hz frequency range with approximately 30–90° phase shift between leader and follower. This means that the head motion of the Follower follows the Leader by 30–90° of phase. Note also that the blue circled areas in the top half of Fig. 4c reflect the hypo-coherence at high frequencies described above, confirming that this pattern can also be seen in a different analysis.

**Fig. 5:**
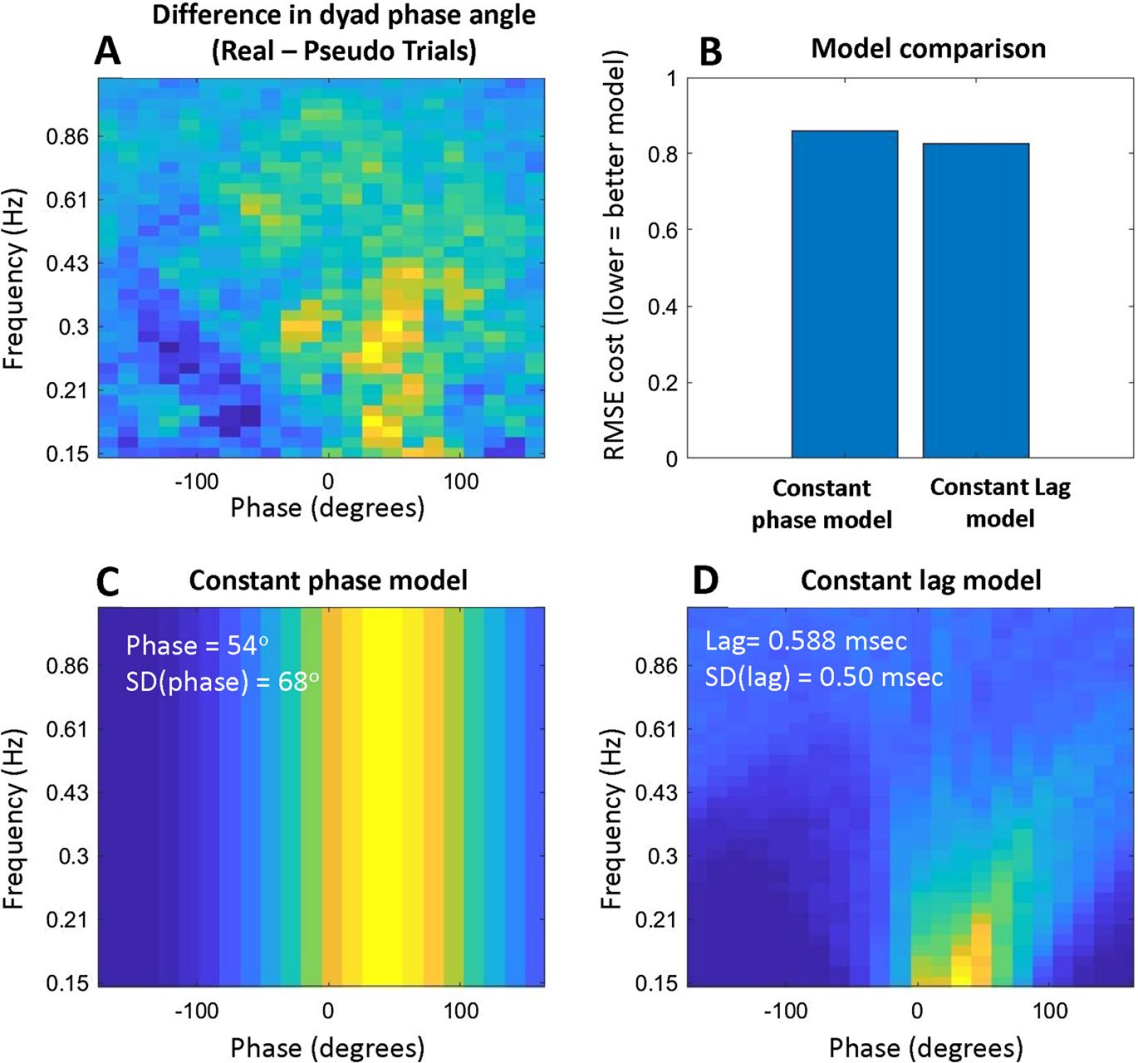
Modeling the phase-frequency relationship. **a** The same data as the lower part of Fig. 4c, i.e. the difference in dyads’ average phase relationship between real and pseudo trials. This data was modeled to compare a ‘constant-phase’ model (**c**) with a ‘constant-lag’ model (**d**). Each model was optimized to fit the data and the optimal parameters are given in white text. The constant-lag model gave a better overall fit to the data in terms of the root-mean-squared-error (RMSE) (**b**)

Two different mechanisms could potentially drive the phase effect shown in Fig. 4c. Followers could be in sync with leaders, maintaining a specific phase relationship (e.g., 40° of phase) across a range of frequencies (‘constant-phase’ mechanism). Or followers could lag behind leaders with a fixed delay (‘constant-lag mechanism). The appearance of Fig. 4c, with slightly greater phase shifts for slightly higher frequencies, suggests the latter explanation. To test this formally, we built models of the two potential mechanisms—a constant-phase model and a constant-delay model (see supplementary information). The optimal model outputs are shown in Fig. 5. Note that this figure includes a slightly different phase range, from 0.15 to 0.9 Hz. The root-mean-squared-error (RMSE) of the constant-delay model was lower than the constant-phase model, indicating that this gives a better explanation of the data. The optimal constant-delay parameter of 0.588 s is close to the mean delay of 0.63 s in the cross-correlation analysis of Fig. 4a. Thus, both analyses support the idea that leader-follower head motion can be characterized by a constant-delay model with a delay of approximately 0.6 s.

### Exploring Hypo-coherence in Head Pitch at High Motion Frequencies

In addition to finding coherence at low motion frequencies, we found significant hypo-coherence at high frequencies in the range 2.6–6.5 Hz. Head pitch (nodding) movements in this range are very small and quick, and could potentially represent a different social signal from nodding in the low-frequency range. To understand the hypo-coherence pattern in our data, it helps to clarify what hypo-coherence between two signals actually means. In a cross-wavelet analysis, two signals have high coherence if both have energy at the same frequency (Issartel et al. 2014), regardless of the phase relationship between the two signals. Hypo-coherence in a wavelet analysis means that two people are moving at different frequencies, for example, when one moves at 2.5 Hz, the other moves at 3.5 Hz. It is not the same as anti-phase coherence, where two people move at the same frequency but out of phase which each other. We find that this hypo-coherence is reliably present in the dialogue phase of the conversation (Figure S1), indicating that it is not limited to the more artificial context where one participant is not allowed to speak. It is also not driven by a difference in the global power of the head motion signal between the participants (Figure S1). Rather, there must be a more subtle change in behavior.

**Fig. 6:**
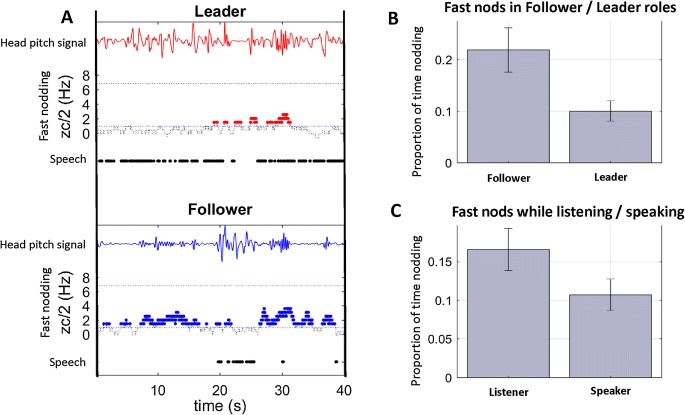
Detection of fast nods and their relationship to speech. **a** A sample timeline from a Leader and Follower in the same trial, illustrating the head pitch signal (top of plot), the output of the fast-nod detection algorithm (zc/2, x-axis) and whether that person is speaking or not (bottom of plot). Fast nods (colored dots) are more common when a participant is listening. This is seen when comparing fast-nods categorized by the role of Leader versus Follower over a whole trial (**b**) and also when comparing the rate of fast-nods when an individual is speaking or not-speaking (determined from audio data for each time point within a trial) in **c**

To explore this, we labeled the hig- frequency motion in the head pitch signal as ‘fast nods’ and build a fast-nod detector to determine when and why this behavior occurs. The fast-nod detector estimates the dominant frequency in head pitch using a zero-crossing algorithm (see Supplementary Information). Using this detector, we coded each 1-second window in our data as containing a fast nod from the leader, the follower, both, or neither. Figure 6a illustrates the fast nods detected in dialogue from one sample trial, classified by Leader and Follower roles. We found that followers spend 22% of the trial engaged in fast-nod behavior while leaders spend only 10% of the trial engaged in this behavior, and this difference is significant (*t*(25) = 5.83, *p *< .00001, effect size *d* = 1.394; Fig. 6b). In terms of classifier precision (where precision = nodding time as follower/total nodding time), 67% of any reported fast nods are performed by a person who is in the follower role.

This effect suggests that fast nodding is probably related to listening behavior, since the role of the Follower is to listen to the Leader. However, it is possible to examine the relationship between listening and fast-nodding in more detail by using audio recordings of each trial. We recorded audio signals from each participant onto the left and right channels of a single audio file, and thus can classify who was speaking at each time point in the trial using a simple thresholding of signal energy in the left and right channels. We then test, for each trial, how the speaking behavior of X (speaking/not speaking) predicts the fast-nodding behavior of Y (nodding/not nodding) and vice versa, irrespective of Leader/Follower roles. We find that participants are more likely to be nodding when their partner is speaking than not (*t*(23) = 4.08, *p *< .001, effect size *d* = 0.843). Figure 6c plots this difference in terms of the proportion of fast nods Y made while their partner X was speaking (17%) or not speaking (11%). The precision of this classifier is a high 94%. This means that if one person is fast-nodding, it is extremely likely that their conversation partner is speaking.

## Discussion

This paper presents a detailed analysis of a rich dataset describing head motion of dyads during a structured conversation. Using high-resolution recordings and robust analysis methods, we find evidence for important motion features which have not previously been described. First, real pairs of participants show head pitch (nodding) coherence at low frequencies (0.2–1.1 Hz) and this coherent motion has a timing lag of 0.6 s between leader and follower. Second, significant hypo-coherence in head pitch is seen at high frequencies (2.6–6.5 Hz frequency band), with evidence that this is driven by fast-nodding behavior on the part of listeners (not speakers). We consider what this means for our models of human social interaction and for future studies.

### Low-frequency Coherence

Our data reveal low-frequency coherence between 0.2 and 1.1 Hz with a time lag around 600 ms between the designated Leader (main speaker) and Follower (main listener). This pattern is consistent with many early reports of mimicry (Condon and Ogston 1966) as well as more recent motion capture studies (Fujiwara and Daibo 2016; Schmidt et al. 2014). Our key innovation here is obtaining a precise measure of the time lag in spontaneously occurring mimicry behavior (i.e., not instructed or induced through a task), which we find to be close to 600 ms. Knowing this value is critical in distinguishing between the different possible neurocognitive models of mimicry.

A lag of around 600 ms suggests that interpersonal coordination of head movements is not driven by an anticipatory mechanism which perfectly synchronizes the motion between two people. Such mechanisms can be seen in tasks involving musical rhythms (Konvalinka et al. 2010) but do not seem to apply here. The difference between our data and previous reports emphasising the importance of synchrony (Hove and Risen 2009; von Zimmermann et al. 2018) could be due to two different factors. First, we recorded head movements while others recorded hand or finger movements, and there may be different mechanisms governing different body parts. Second, we placed participants in the context of a structured conversation rather than the context of dance or choreographed movement, and participants may use different coordination mechanisms in different contexts.

We also do not see strong evidence for interpersonal coordination at long time scales (2–10 s lag) as reported in previous studies of behavioral mimicry (Chartrand and Bargh 1999; Stel et al. 2009). The slowest mimicry in our data can be seen at the lowest end of the frequency range, for example 0.2 Hz actions at 90° of phase implies a 2 s delay and can be seen at the bottom of Fig. 5c. Previous reports of long-lag mimicry present a potential conundrum for neurocognitive models of this behavior, because they might require a memory mechanism in addition to a mimicry mechanism; that is, if in real-world contexts, people see an action from their partner, hold it in mind, and then produce it up to 10 s later, this implies a role for short term memory. However, the dominant pattern in our data is for faster mimicry behavior, which can be best explained by a constant-lag model with a lag of around 600 ms. This pattern of data is consistent with a reactive mechanism in which one person sees an action and does a similar action around 600 ms later. There is no need for either prediction or memory of the other’s action in explaining the patterns of behavior we see. Thus, our finding provides support for the claim that mirror neuron systems (Iacoboni et al. 1999) or general visuomotor priming mechanisms (Heyes 2011) are sufficient to implement mimicry of real-world head movements in conversation. It also suggests that, at least for head movements, the long-lag actions which have previously been reported (Chartrand and Bargh 1999; Stel et al. 2009) may not be the dominant form of mimicry behavior. However, the relatively short trials in our study (90 s) and the focus on the 0.2–8 Hz frequency range means that we could have missed long-lag actions in our analysis. Overall, the high-resolution data recordings in the present dataset allow us to precisely parameterize real-world interactive behavior and thus link it to cognitive models.

### High-frequency Hypo-coherence

An unexpected finding in our data was a pattern of high frequency (2.6–6.5 Hz) hypo-coherence, whereby the two participants show systematically less-than-chance coherence in their head motion. Given the wealth of evidence that people spontaneously coordinate other movements (Bernieri et al. 1994; Grammer et al. 1998; Ramseyer and Tschacher 2010; Schmidt et al. 2012), and the focus on synchronous or coherent behavior in the literature (Chartrand and Bargh 1999; Hove and Risen 2009), it was rather surprising to see active decoupling of head movements at typical frequencies for conversation.

The hypo-coherence we report is not the same as coherence in anti-phase, where two people move at the same frequency but out of phase; here, participants do not move at the same frequency at all. Further exploratory analysis shows that hypo-coherence is tightly linked to each person’s speaking or listening status. We found that leaders (who mainly speak) and followers (who mainly listen) nod with similar energy between 2.6 and 6.5 Hz when data is averaged over whole trials (see Supplementary Information Figure S1C and S1D). However, there also a specific fast-nod pattern at 2.6–6.5 Hz which occurs in the person who is listening (Fig. 4). A simple zero-crossing estimation of dominant frequency in this range allowed us to classify a participant as the designated follower with 67% precision, and as listening to their partner speaking (based on audio recordings) with 94% precision. This result implies that fast-nodding is a complementary action, which is performed by one member of a dyad when the other performs something different. The fact that we can find both fast-nodding and reactive mimicry at different movement frequencies in the same data signal emphasizes the importance of detailed motion recordings and supports the idea that interpersonal coordination involves more than just mimicry.

It is likely that fast-nodding reflects a communication back-channel (Clark 1996), whereby the listener is sending a signal of attentiveness and engagement to the speaker. Many previous studies also report a ‘nodding’ backchannel in human communication (Kendon 1970). However, our result is novel because this is not a slow nod that might be easy to see in a video recording, but is a very small quick head motion, occurring at roughly the same frequency as human speech. This kind of nodding is visible but very subtle, and we would not expect a casual observer or the people interacting to be aware of it, although we have not tested this in the present study. We are aware of one previous report which describe a ‘Brief fast repeated downward movement’ of the head as a form of backchannel (Poggi et al. 2010) but we are not aware of any more detailed work on this behavior. To test whether fast-nodding could be a communication back-channel, further studies would need to determine if listeners produced more fast-nodding when with another person (compared to alone) and if speakers can detect the fast-nodding behavior (on some level).

One way to explore the role of fast-nodding is to see if it differs between the monologue and dialogue segments of the task. Other studies suggest that gaze coordination differs between monologue and dialogue (Richardson et al. 2007), so if there are also differences in nodding, that might help us understand this behavior. An analysis of the dialogue only segment replicates the finding of reliable fast-nodding (Supplementary Information Figure S1B), but this pattern was only weakly present and did not pass our statistical threshold in the monologue only segment (Supplementary InformationFigure S1A). However, a direct t-test comparing the monologue and dialogue segments of the tasks did not reveal any differences between them (Supplementary Information S1C). It is possible that the weaker effect in the monologue data arises because there is less data available (30 s per trial versus 60 s per trial) and thus less power overall. Future studies could examine the role of conversational context in more detail.

One possible criticism of our fast-nodding result is that it is not easy, voluntarily, to produce a small head nod at 2–5 Hz—certainly not at the 5 Hz end of this frequency band. We agree that this behavior cannot easily be produced on command, but that is true of many other socially meaningful signals including genuine (Duchenne) smiles (Ekman et al. 1990; Gunnery et al. 2013) and genuine laughter (Lavan et al. 2016). The lack of a voluntary pathway for a particular behavior does not mean that this behavior cannot have an important role in social signaling. Rather, it makes that behavior hard to fake and means that it may have more value as an honest signal of listening/attentiveness (Pentland 2008). Further studies will be required to test this.

### Limitations

This paper presents a detailed analysis of head-nodding behaviors in a structured conversation task, but there are some limitations to our interpretation. First, our full analysis focus only on head pitch data (nodding) and not roll and yaw. Our focus on just one type of head movement was driven by our pilot data showing that effects were most robust for nodding and we pre-registered our analysis pathway to document this decision. Second, we examined nodding in just one task context—a structured picture description task with 30 s of monologue followed by 60 s of dialogue. We found similar patterns of behavior across the monologue and dialogue segments (see Supplementary Information Figure S1), but it would be useful for future studies to examine a wider range of conversation types. We do not yet know if the effects we find here are generalizable across different styles of conversation include more natural situations. Similarly, we examined only head motion, while mimicry has also been reported in gesture, posture, facial actions, and vocal features. Further studies will be required to determine if the features we find here are also present in other nonverbal behaviors.

Finally, a number of studies have examined how mimicry relates to factors such as liking or the success of therapy (Ramseyer and Tschacher 2011; Salazar Kämpf et al. 2018) using an individual differences approach. In the current study, we collected some basic questionnaire data on liking but did not analyze this in detail. This is because power analyses show that sample sizes of the order of 100–200 participants are required for meaningful analyses of individual differences. With the complex technical demands of running a motion capture study, it was not possible to collect a sample of that size and thus we do not investigate individual differences in detail.

### Future Directions

Our project opens up many future directions for further studies. In particular, it is important to know if and how the social signals we detect here—mimicry and fast nodding—are related to conversational outcomes. For example, does fast nodding predict how much one person remembers of the other person’s speech, as has been found for synchrony (Miles et al. 2010)? Does reactive mimicry predict how much people like each other after the conversation (Salazar Kämpf et al. 2018)? There are several different ways in which these questions can be addressed. One option is to include post-conversation questionnaires in studies like the present one and to test if features of the motion coherence in the conversation relates to how much the participants like each other afterward. We did not examine this in the present study because a robust test of how natural conversational behavior relates to liking or personality traits also requires much larger sample sizes (Ramseyer and Tschacher 2014; Salazar Kämpf et al. 2018) than the present study. It is also often hard to rule out confounds in correlational studies of this type.

A second approach is to impose experimental manipulations before the conversation, such as giving participants a goal to affiliate or setting up particular expectations about their partner, for example, as someone who is antisocial or an outgroup member etc. (Lakin and Chartrand 2003). We can test if these manipulations alter the use of mimicry and fast-nodding behaviors during a conversation task, and thus understand how different motivational factors change social coordination behavior. Similarly, we can explore how conversation behavior varies across different clinical groups including participants with autism or other psychiatric conditions (Ramseyer and Tschacher 2014). This might allow a more detailed understanding of what aspects of a real-world conversation differ across different clinical conditions and how this might be used for diagnosis or measures of treatment effects.

A third option, which provides the greatest experimental control, is to impose the mimicry and fast-nodding signals on virtual reality characters engaged in a social interaction with a participant, and test how the participant responds to the character. For example, do participants prefer to interact with a virtual character who mimics their head movements? Previous data give ambiguous results (Bailenson and Yee 2005; Hale and Hamilton 2016) but have not used virtual characters whose actions precisely reflect the contingencies found in natural conversation. Datasets such as the one presented here will allow us to build more natural virtual characters, who mimic head movements with a 600 ms delay (but not all the time) and who show fast-nodding behavior when listening. We can compare these to virtual characters who show different conversation behaviors, and test how they engage in the conversation or which character participants prefer to interact with. For example, recent work using the approach showed that blinks can act as a back-channel (Hömke et al. 2018) and the same might apply to fast-nods. This VR method provides the most robust test of how different coordination patterns in conversation drive liking or affiliation in an interaction.

It will also be important to understand more about the neural and cognitive mechanisms underlying real-world conversation behavior. The high-resolution recordings in the present study allow us to pin down the timing of naturally occurring mimicry to around 600 ms, which implies the involvement of a mirror neuron mechanism. It would be possible to use the next-generation of wearable neuroimaging systems such as functional near infrared spectroscopy (fNIRS) to capture patterns of brain activation across the mirror neuron system while people engage in structured or natural conversation tasks, and thus relate mimicry behavior directly to brain mechanisms. Similarly, it would be possible to test how measures of inter-brain coherence (Hirsch et al. 2017; Jiang et al. 2015) relate to the behavioral coherence reported here. This will bring the field substantially closer to the study of real-world second person neuroscience (Schilbach et al. 2013).

### Conclusions

This paper describes a rich dataset of head movements in structured conversations and provides new insights into the patterns and mechanisms of social coordination. We show coherent head motion at low frequencies (0.21–1.1 Hz) with a time lag around 600 ms. This implies a simple reactive visuomotor mechanism which is likely to be implemented in the mirror neuron system. We also show reliable hypo-coherence at high frequencies (2.6–6.5 Hz) which may be linked to fast-nodding behavior on the part of the listener. The present study builds on a growing literature exploring interpersonal coordination through automatic motion capture and spectrum analysis. Such detailed analysis of the ‘big data’ of human social interactions will be critical in creating a new understanding of our everyday social behavior and the neurocognitive mechanisms which support it.

## Electronic Supplementary Material

Below is the link to the electronic supplementary material.

Supplementary material 1 (PDF 564 kb)

## Data Availability

Anonymized data & analysis code will be uploaded to OSF when this manuscript is accepted.
