# Supplementary material for: Are You on My Wavelength? Interpersonal Coordination in Dyadic Conversations
Source: J Nonverbal Behav. 2019 Oct 15;44(1):63–83. doi: 10.1007/s10919-019-00320-3 (PMC7054373; doi:10.1007/s10919-019-00320-3)
Supplement: Supplementary file 1 — Supplementary material 1 (PDF 564 kb) [file 10919_2019_320_MOESM1_ESM.pdf]

Supplementary information for:

Are you on my wavelength? Interpersonal coordination in naturalistic conversations

Jo Hale*, Jamie A Ward*, Francesco Buccheri, Dom Oliver, Antonia F de C Hamilton

1) Are effects similar across monologue and dialogue?

The analysis described in the main text used the full-trial data, with both monologue and dialogue analysed together. To explore potential differences between monologue and dialogue, we repeated the wavelet coherence analyses of head pitch data, separated into these two trial sections. Results are shown in Figure S2, A. We find that the monologue dataset shows low frequency coherence but not high frequency hypo-coherence (Fig S1 A & D). The dialogue dataset shows both effects (Fig S1, B and E). This means that the results we report are not an artefact of the more unnatural monologue situation. A direct test of the difference between monologue and dialogue (Fig S1, C and F) does not show any results passing FDR corrected significance thresholds.

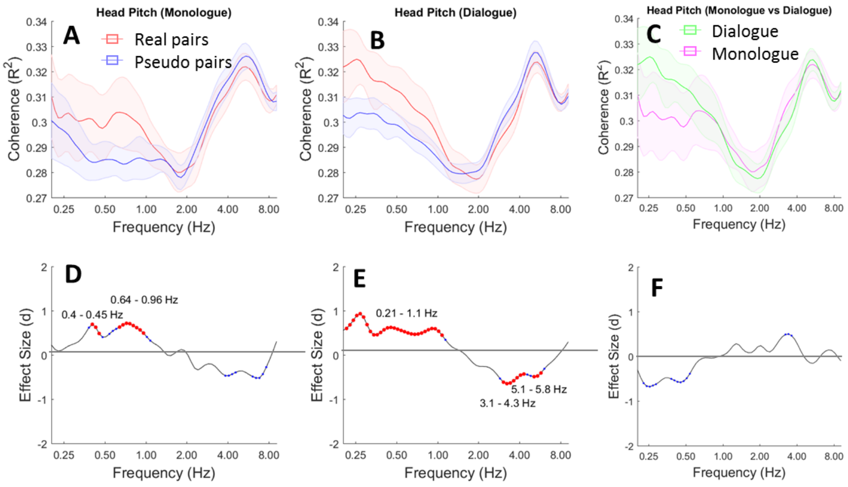

Figure S1. Head pitch coherence plotted for monologue and dialogue modes of conversation separately. A and D illustrate the coherence effect for the Monologue segments. B and E illustrate the coherence effect for the dialogue segments. C and F provide a direct comparison between the monologue and dialogue, showing that there are no FDR corrected differences between these. In all cases, red dots indicate effects which are significant at p<0.05 FDR corrected.

2) Do global differences in signal power drive the hypo-coherence effect?

This analysis aimed to determine if global differences in signal power could drive the hypo-coherence effect. For example, if one participant moved much less than the other at 3Hz, then there would be no power to drive coherence at 3Hz. As we have used a strict, within-dyad comparison between real trials and pseudo-trials, it is unlikely that power differences drive our effects, because

data with similar power occur in both the real and pseudo trial analyses. To double-check this, we calculated power spectral density (PSD) over the whole trial (without breaking the data into wavelets) to check for global differences in signal power between participants and trial sections. For each participant and trial, we calculated the PSD using Matlab's *pwelch* function. Then we averaged data according to the participant's role as Leader or Follower in that trial. This allowed us to determine if there were overall differences in participants' movement behaviour between the leader / follower roles and monologue / dialogue sections (Figure S3).

In the monologue case (Fig S2A&B), we show that leaders have more power than followers at low frequencies (the range where we identified low-frequency mimicry). This corresponds to the fact that leaders do all the talking during monologue. But at higher frequencies, and throughout most of the dialogue case, as shown in Fig S3B&D, the leader and follower power levels are evenly matched.

In all cases, red dots indicate effects which are significant at p<0.05 FDR corrected.

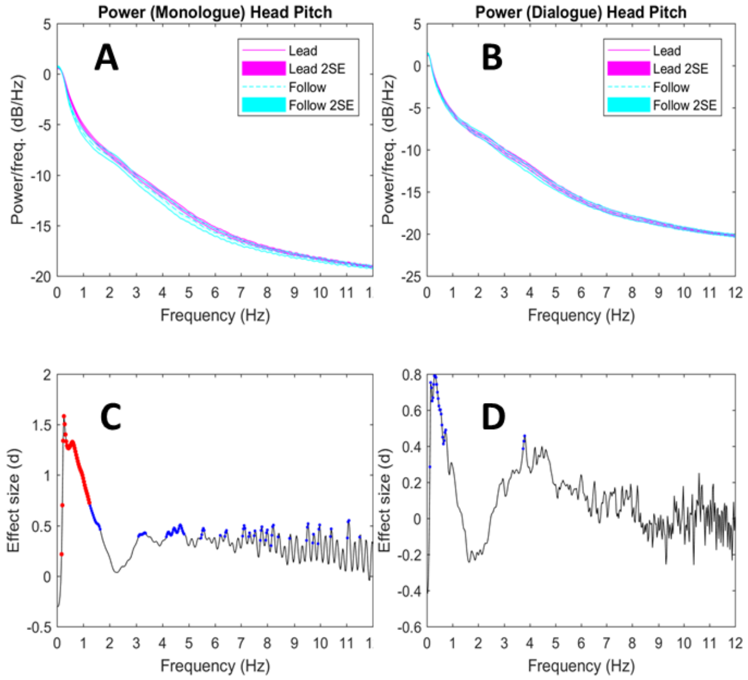

Figure S2. A and B illustrates the power at each frequency in the leader and follower data (for monologue and dialogue respectively), and C and D illustrates the effect size for a difference in power between the two roles (for monologue and dialogue).

3) Exploring the characteristics of low frequency mimicry (Figure 4)

The wavelet coherence analysis revealed interpersonal coherence at low frequencies associated with mimicry in our data. To explore the time-lags involved, we cross-correlated the raw head-pitch data from the Leader and Follower in each dyad for a range of time lags between -4 and 4 seconds using Matlab's xcorr function. We did this for real trials and pseudo trials, then averaged over trials and dyads and used paired t-tests with FDR correction to contrast the real and pseudo trial data (Fig 4 A and D).

As well as exploring time-lag, we explored the phase-difference between the Leader and Follower. When calculating wavelet coherence, it is possible to obtain information on both the coherence level and the phase difference between the two signals. Phase can only be meaningfully interpreted when there is positive coherence – that is, two signals are active in the same frequency range. For this reason we only store phase data when coherence meets a minimum threshold; here we choose a mid-range threshold of 0.5. For every dyad and trial, we calculated the phase difference between Leader and Follower (Fig 2G), and then thresholded this image to show only data points with a coherence over 0.5. For each frequency band, we then counted the number of supra-threshold points falling into each of 24 phase bins from -180° to 180°. This collapses the data over time and reveals the distribution of phases, and we can plot this data as a phase-frequency histogram (Fig 2H). We then average phase-frequency histograms over all trials and all dyads for both real trials and pseudo trials (Fig 4B and E). The difference between phase-frequency plots for real and pseudo trials (Fig 4C) reveals the frequency bands at which participants are in phase with a specific lag (yellow areas) and where less data is present than chance (blue areas). Thresholds on this map were created with paired-sample t-tests.

4) *Modelling the phase-frequency histograms (Figure 5)*

We aimed to test if the phase-frequency relationship seen in the lower part of Fig 4B (repeated in Fig 5A) is generated by a constant-phase mechanism or a constant-lag mechanism. To do this, we built two simple generative models, one for each mechanism. The constant-phase model had two parameters – phase lag and variability – and was modelled as a Gaussian distribution of phases about a fixed mean (Fig 5C). The constant lag model also had two parameters – time lag and variability – and was modelled by sampling individual trials, offsetting the Leader movement by the time lag relative to the Follower, then calculating the wavelet coherence and phase-frequency histogram for that sample trial. This process was repeated for 416 iterations (because the original dataset had 16x26 = 416 trials) using time-lags drawn from a Gaussian with mean & variability specified by the model parameters. The results of the 416 iterations were averaged to give the final result (Fig 5D). For each model, we used Matlab's fminsearch function to find the parameter values which gave the best fit between the model and the data, that is, to minimise the root-mean-squared error (RMSE) between the generated data (Fig5C or D) and the original data (Fig 5A). The model outputs shown in Fig 5 C and D represent the model using the optimal parameters. Comparing the RMSE values for the two models shows that the constant-lag model has a better fit to the data (Fig 5B). This implies that the cognitive mechanisms generating mimicry of head nods act with a constant lag of around 0.588 msec.

5) *Exploring high frequency hypo-coherence in head motion*

Our wavelet analysis highlighted the 2.6-6.5Hz frequency range as an interesting band where one participant might engage in a fast-nodding behaviour while the other does not show coherent head motion. To explore this, we developed a 'fast nod detector' algorithm to test which participant shows fast-nods and when. We defined fast nods in this context as head-pitch movements with a dominant frequency within the wider range of 1.5 to 8 Hz. This range is selected (rather than our significant findings of 2.6 to 6.5 Hz) to account for the approximate nature of our detector, and covers the range of frequency bands in Fig 3D with an effect size less than zero. To detect these frequencies we performed thresholding on an estimate of the dominant frequency obtained from the zero-crossing rate (ZC). The ZC algorithm works by counting the number of times a signal crosses the zero (or window mean) within a given window, and is implemented here as follows:

1. slide a 2 second window across the pitch data,
2. high-pass filter by removing the window mean,
3. count the number of times the signal makes a zero crossing in one second (ZC),
4. calculate the zero-crossing rate as an approximation of the frequency (ZC/2 approximates frequency in Hz),
5. select those time windows where the approximate frequency falls within the wider range of 'fast nods' (between 1.5 and 8 Hz).

Dominant frequency can be computed by other means, like Fast Fourier Transform (FFT), however the ZC method has the advantage of simplicity and ease of implementation for future real-time execution. (One of the planned follow-on projects from this work is to implement an interactive virtual agent that can detect and respond to the head nods of human interlocutors in real time.)

The output of the fast-nod detector for each participant is a vector of 0/1 for each 1-second time window in each trial marking the presence / absence of a fast nod from that participant. Examples are shown in Figure 6A. Averaging this vector gives an estimate of the rate of fast-nodding for that participant, and a paired t-test was used to compare rates between trials where that participant had a Leader role and trials where they had a Follower role (Figure 6B). In addition, a speech-detector which thresholded the audio data was used to mark each timepoint in the data as 'X speaking', 'Y speaking' 'Both speaking' or 'neither speaking'. Note that audio quality was too low to detect who was speaking in 2 dyads, so the sample size for this analysis is n=24 dyads. We used this to calculate the rate of fast nods for each participant when speaking and when not speaking, and then used a paired t-test to compare fast-nodding rates between Speaking and not-speaking phases within trials. We can characterise the performance of the detector in terms of precision and

recall. Precision measures the proportion of nods that occur during Following/not speaking against all detected nods. Recall measures the proportion of nods that occur during Following/not speaking against all periods marked as listening/following (Fawcett, 2003). Results of this analysis are shown in Figure 6.
